# Serological and molecular analysis of henipavirus infections in synanthropic fruit bat and rodent populations in the Centre and North regions of Cameroon (2018–2020)

**DOI:** 10.1186/s12917-025-04530-4

**Published:** 2025-02-24

**Authors:** Cyrille Mbanwi Mbu’u, Pierre Gontao, Abel Wade, Maren Penning, Balal Sadeghi, Aristid Ekollo Mbange, Matthew LeBreton, Sylvain Leroy Sado Kamdem, Franziska Stoek, Martin Hermann Groschup, Wilfred Fon Mbacham, Anne Balkema-Buschmann

**Affiliations:** 1 Department of Microbiology, Faculty of Science, University of Yaoundé 1, Yaoundé, Cameroon; 2 Biotechnology Centre-University of Yaoundé 1 (BTC-UY1), Laboratory for Public Health Research Biotechnologies (LAPHER Biotech.), Yaoundé, Cameroon; 3 Department of Biological Sciences, Faculty of Science, University of Maroua, Maroua, Cameroon; 4 National Veterinary Laboratory (LANAVET), Yaoundé, Cameroon; 5 Friedrich-Loeffler-Institut (FLI), Institute of Novel and Emerging Infectious Diseases (INNT), Greifswald-Insel Riems, Greifswald, Germany; 6 University of Ngaoundere, Institute of Technologies, Ngaoundere, Cameroon; 7 Mosaic, Yaoundé, Cameroon; 8 Faculty of Natural and Agricultural Sciences, The Northwest University, North-West University, Potchefstroom, South Africa; 9 Centre for Health Implementation and Translational Research (CHITRES), The Fobang Institutes for Innovation in Science and Technology (FINISTECH), Box 8094, Yaoundé, Cameroon

**Keywords:** Henipaviruses, Zoonotic disease, Fruit bats and rodents, Bead-based multiplex binding assay and ELISA, Centre and North regions of Cameroon

## Abstract

**Background:**

Bats and rodents have been identified as reservoirs for several highly pathogenic and zoonotic viruses including henipaviruses, a genus within the *Paramyxoviridae* family. A number of studies have revealed the circulation of henipaviruses at the wildlife-human-livestock interface in Cameroon. In this study, we describe the molecular analysis as well as the development and evaluation of a Bead-based Multiplex Binding Assay (BMBA) using an in-house Indirect Enzyme Linked Immunosorbent Assay (ELISA) to confirm the detection of henipavirus infection in wildlife species.

**Results:**

A total of 600 fruit bats and 600 rodents were sampled between March 2018 and June 2020. Samples were analyzed using a semi-nested RT-PCR assay followed by sequencing of the PCR fragments. Transudates (754) were screened for the presence of henipavirus-specific antibodies in a BMBA and confirmed by ELISA using Hendra virus (HeV), Nipah virus (NiV) and Ghana virus (GhV) glycoproteins expressed in *Leishmania tarentolae*, and commercially available HeV G and NiV G glycoproteins.

Henipavirus-specific antibodies were detected in 19/531 (3.6%) bat transudates screened by BMBA and confirmed by ELISA. Seroprevalence rates in the Centre and North Regions were 12/291 (4.1%) and 7/240 (2.9%) respectively. All rodents and shrews were serologically negative. Henipavirus RNA sequences were not detected in any of the samples screened in this work.

**Conclusion:**

This study provides further data supporting the circulation of Henipaviruses in fruit bats (*Eidolon helvum*) which are roosting and reproducing in proximity to human and livestock populations in the Centre and North Regions of Cameroon. This also establishes the first detection of Henipavirus specific antibodies in *Eidolon helvum* populations in the North Region of Cameroon.

**Supplementary Information:**

The online version contains supplementary material available at 10.1186/s12917-025-04530-4.

## Background

At least 75% of emerging infectious diseases (EIDs) affecting humans originate from animals [1]. Of the 1400 known human diseases, 60% are animal-borne [2], while 80% of known biological weapons have a zoonotic origin [3]. Viruses and prions account for 25% of EIDs [4] and over the past few decades, an increasing proportion of newly detected human pathogens were viruses [5]. Viral pathogens (RNA viruses especially) represent a threat to public and animal health owing essentially to their often-segmented genomes, and often-high rates of nucleotide substitution coupled with the lack of proofreading ability of viral polymerases to correct mutational errors. These processes drive the rapid evolution of the virus and its adaptability to new hosts upon transmission, including humans [4].

Bats are reservoir hosts for a good number of emerging zoonotic viruses including filoviruses, paramyxoviruses, coronaviruses, bunyaviruses and rhabdoviruses [6–9]. Moreover, fruit bats of the sub-order *Yinpterochiroptera*, genus *Pteropus* [10], found in South-East Asia and Australia as well as the straw-coloured fruit bat (*Eidolon helvum* and *Eidolon dupreanum*) which are widely distributed in Africa and other countries including Saudi Arabia [11] and Yemen [12], have been shown to be natural hosts for henipaviruses and other paramyxoviruses [13–17]. Rodents and shrews are among the reservoir hosts for diverse human viral pathogens from families such as *Arenaviridae*,* Hantaviridae*,* Phenuiviridae*,* Reoviridae*,* Togaviridae*,* Coronaviridae*,* Picornaviridae*,* Paramyxoviridae* and *Flaviviridae* [18–22].

The most relevant members of the genus *henipavirus* originate from fruit bats and include Nipah virus (NiV), Hendra virus (HeV), Cedar virus (CedV) and Ghana virus (GhV) while recently, Angavokely (AngV) related-RNA was discovered in fruit bats (*Eidolon dupreanum*) in Madagascar [17]. Meanwhile, Mojiang virus (MojV) is rodent-borne (*Rattus flavipectus*) [23–25], and Gamak virus (GakV) as well as Daeryong virus (DarV) have been identified by molecular analysis in shrews in the Republic of South Korea [26]. Most recently, the zoonotic Langya virus (LayV) which has been identified in humans with history of animal exposure and shrew populations from China was revealed to be closely related to MojV [27].

Bat-borne emerging zoonotic paramyxoviruses such as NiV and HeV are listed by the World Health Organization (WHO) as priority pathogens [28]. HeV and NiV have been widely investigated in Australia and South-East Asia in the last three decades due to their high mortality and morbidity rates in both human and animal populations. Both viruses may cause severe respiratory illness, encephalitis and neuro-degenerative diseases mainly in humans, pigs and horses, with case-fatality rates in humans frequently reaching 75% or even higher [29–31]. Indeed, about 63 natural HeV spillover events have been reported in Australia causing 105 deaths in horses and 4 deaths in 7 confirmed human cases. In South-East Asia, NiV has caused more than 700 human deaths [32]. Transmission of the virus occurs either by direct or indirect contact, and livestock species such as pigs and horses serve as amplifying hosts for NiV and HeV respectively [33, 34].

Moreover, substantial serological and molecular evidence revealed the distribution of henipaviruses in wildlife (bats), livestock (pigs and horses) and human populations in Africa despite no reports of clinical cases or sporadic epidemics in this part of the world [35]. Henipaviruses and other paramyxoviruses have been detected in African bats, rodents, shrews and livestock from Sub-Saharan African countries such as Ghana and Nigeria in West Africa; Republic of Congo (RoC), Democratic Republic of Congo (DRC) and Gabon in Central Africa; Annobón Island in the Gulf of Guinea; Zambia, Rwanda, Uganda and Tanzania in East Africa; Madagascar, an island off the Southeastern coast of Africa, as well as in South Africa [16, 20, 35–37].

There is a paucity of data on the serological and molecular evidence of henipaviruses at the human-wildlife interface in Cameroon. An earlier study revealed henipavirus-specific antibodies in fruit bats and humans at incidence rates of 48% and 4% respectively, indicating spillover of the virus into human populations in Cameroon [38]. In contrast, no data are available on a possible prevalence of henipavirus infections in rodents and shrews in Cameroon. A number of surveillance studies have been conducted on fruit bat populations (*Eidolon helvum* and *Epomophorus gambianus*) in the South-West Region of Cameroon by metagenomics. These studies revealed a diversity of viruses including papillomaviruses [39], sapoviruses [40], rotaviruses [41], picornaviruses [42], picobinaviruses and bastroviruses [43] as well as coronaviruses from bats, rodents and shrews [44].

Indeed, the diagnosis of henipavirus infection is challenged by the absence of validated commercialized serological and molecular kits. However, a number of in-house serological assays ranging from Enzyme Linked Immunosorbent Assays (ELISAs) to microsphere immunoassays (Bead-Based Multiplex Binding Assay (BMBA)) have been developed to detect henipavirus antibodies in experimental models and field samples [45–49]. Most of the serological assays are based on the recombinant G glycoprotein from NiV and HeV sharing a percentage sequence identity of 83% and may explain why antibodies raised against these viruses cross-react [45, 50]. Even though the serum neutralization test (SNT) remains the gold standard for henipavirus detection in serology, it requires working in a high containment laboratory (BSL4) of which on the African continent only one exists in South Africa and none exists in Sub-Saharan African countries. Therefore, the need to establish a simple, cost effective and reliable (sensitive and specific) serological assay for the serosurveillance of henipavirus infection in wildlife populations under low containment conditions is imperative. Thus, in this study, we describe the molecular analysis as well as the development and evaluation of a BMBA using an in-house indirect ELISA test to confirm the detection of henipavirus infection in wildlife species such as bats and rodents in the Centre and North Regions of Cameroon.

## Results

### Sampled bat population

**Fig. 1:**
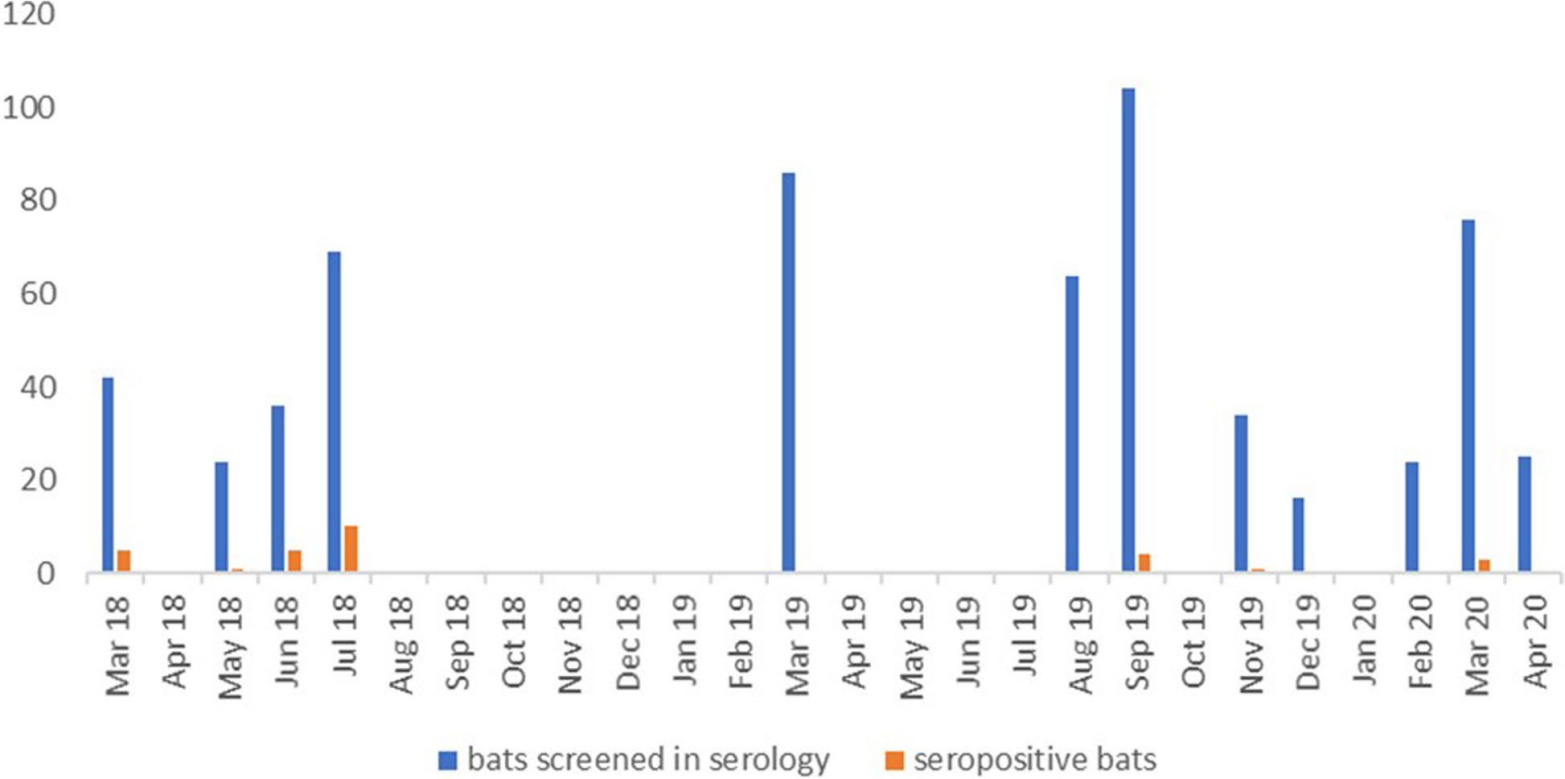
Animals screened by serological analysis and seropositive bats per collection months (North and Centre regions)

In this study, a total of 600 bats were sampled in Cameroon, out of which 300 (50%) originated from the Centre Region, and 300 (50%) from the North Region. Due to the seasonal presence of *Eidolon helvum* bats in the sampled areas, the samples were collected in the months of March, May, June, July, September, November and December (Fig. 1 and Suppl. Table 3). From these animals, 531 transudates were available for serological analysis, among which 291 (54.8%) originated from the Centre Region while 240 (45.2%) were from the North Region of Cameroon. The majority of tested bats were adults (406; 76.5%), followed by sub-adults (110; 20.7%), and lastly juveniles (15; 2.8%). Moreover, there were slightly more females (296; 55.7%) than males (234; 44.1%) while the gender of one bat remained unidentified due to its tender age. Finally, all bats but one, which was identified as *Hypsignathus monstrosus*, were morphologically identified as *Eidolon helvum* (Table 1).

**Table 1:** Characteristics of bats sampled in the Centre and North regions

	Number of sampled bats	Proportion of sampled bats (%)	Number of bats screened in serology	North Region	Number of sampled bats	Proportion of sampled bats (%)	Number of bats screened in serology
Centre Region -Yaounde				North Region-Garoua			
Sampling sites				Sampling sites			
Cascades- MINESUP	64	21.3	61	Sodecoton Guider	29	9.7	29
GP Melen	18	6	15	ENEO Guider	32	10.7	15
MINEPAT	72	24	69	Chefférie Mayo- Oulo	139	46.3	119
Warda	108	36	106	Sodecoton Pitoa	100	33.3	77
Messa	38	12.7	40	/			
**Species**				**Species**			
* Eidolon helvum*	298	99.3	290	* Eidolon helvum*	300	100	240
* Hypsignathus* *monstrosus*	2	0.7	1				
**Age class**				**Age class**			
Adult	225	75	220	Adult	237	79	186
Sub adult	62	20.7	59	Sub adult	56	18.7	51
Juvenile	13	4.3	12	Juvenile	7	2.3	3
**Gender**				**Gender**			
Male	94	31.3	93	Male	182	60.7	141
Female	205	68.4	197	Female	118	39.3	99
Unidentified	1	0.3	1	/			
**Total**	**300**	**100**	**291**	**Total**	**300**	**100**	**240**

From the 300 sampled rodent and shrew samples, 223 transudate samples were available for analysis.

### Serological analysis

Transudates collected in our study were added 0.5% SDS and incubated for 1 h at 56°C to reduce the risk of exposure to zoonotic agents for the staff working with the samples. An experiment with three negative sera and six sera from experimentally immunized animals showed that this treatment did not interfere with the serological analysis for the detection of antibodies against henipaviruses (Table 2).

**Table 2:** BMBA and ELISA for negative and positive serum samples treated for 1 h at 56°C with or without the addition of 0.5% SDS

	BMBA HeV G - SDS	BMBA HeV G + SDS	BMBA NiV G - SDS	BMBA NiV G + SDS	BMBA GhV G - SDS	BMBA GhV G + SDS	ELISA HeV G -SDS	ELISA HeV G + SDS	ELISA NiV G - SDS	ELISA NiV G + SDS	ELISA GhV G - SDS	ELISA GhV G + SDS
Pig negative	57	68	80	93	273	245	0,24	0,30	0,38	0,40	0,69	0,36
Rabbit negative	26	24	24	26	39	37	0,16	0,13	0,05	0,06	0,12	0,06
Bat negative	41	22	62	24	35	30	0,27	0,10	0,15	0,06	0,11	0,05
HeV G rabbit	15104	16050	15457	15813	501	484	3,81	3,85	3,93	3,89	1,28	1,27
HeV G bat	4054	3983	5027	4801	168	151	2,21	1,92	1,95	1,87	0,11	0,08
NiV G pig	12935	12495	16156	15550	4396	4144	4,00	3,75	3,94	4,00	2,25	2,09
NiV G bat	11422	11631	11023	10070	1332	1248	3,73	3,81	3,85	3,92	0,41	0,33
GhV G rabbit	14228	14058	14740	15385	12001	11827	3,77	3,94	3,05	3,83	3,43	3,27
GhV G pig	9310	8915	8949	13930	332	265	3,98	3,97	3,83	3,85	0,44	0,58

The cut-off for the interpretation of results was calculated based as the mean OD values of negative samples plus 3 times the standard deviation (SD). This was done for both assays (BMBA and ELISA), and for each recombinant antigen. Samples where the results were repeatedly above the cut-off value were considered positive. Based on this interpretation, henipavirus-specific antibodies were detected in 19/531 (3.6%) bat transudates screened by BMBA and ELISA which indicated seroprevalences in the Centre and North Regions of 12/291 (4.1%) and 7/240 (2.9%) respectively, as summarized in Table 3.

**Table 3:**
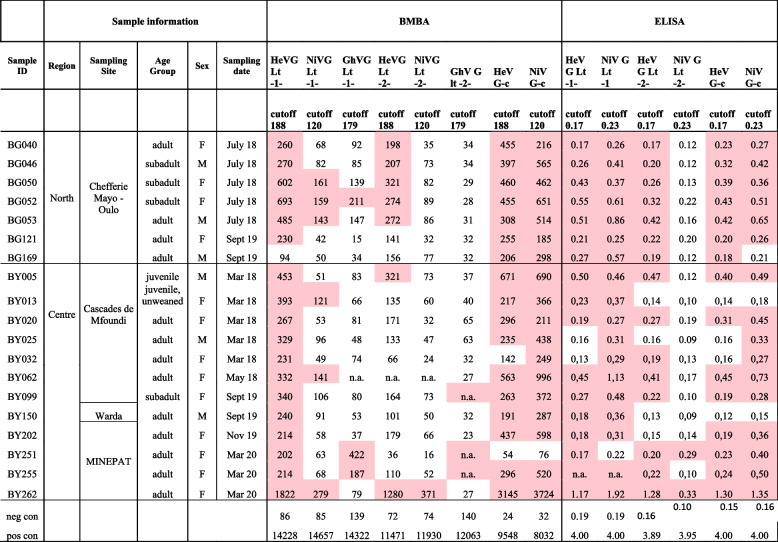
Serological analysis of bat samples. All samples were screened by BMBA using the recombinant proteins expressed in *L. tarentolae* (lt). Reactive samples were repeated in the BMBA using the in-house (lt) as well as commercially available (c) proteins, and in the indirect ELISA using the in-house (lt) proteins as well as commercially available (c) proteins

*NiV G Lt* Nipah virus glycoprotein expressed in* Leishmania tarentolae*, *HeV G Lt* Hendra virus glycoprotein expressed in L. tarentolae, *GhV G Lt* Ghana virus glycoprotein expressed in L. tarentolae, *NiV G-c* Commercially available NiV G, *HeV G-c* Commercially available HeV G, Values under BMBA represent the Median Fluorescence Intensity (MFI) and values under ELISA represent Optical Density (OD)

*-1-* first analysis, *-2-* repeated analysis,*neg con * Negative control, *pos con* Positive control, It needs to be kept in mind that the control samples were sera that were collected after an optimized prime / boost protocol under experimental conditions and immediately stored frozen, while the field samples analyzed here are transudates collected from dead animals of which the timepoint of exposure to the antigen is completely unclear

*Red*:result > cutoff value

Interestingly, antibodies specific to henipaviruses were detected in four different clusters (colonies); one in the North Region (Chefferie Mayo – Oulo) and three in the Centre Region (Cascades de Mfoundi, Warda and MINEPAT). Moreover, henipavirus-specific antibodies were found to be in circulation throughout the sampling period from 2018 to 2020, with clear peaks of reactive samples being found in the months of March, July and September (only 2 / 19 reactive samples (10.5%) were detected in other months (Table 3). We detected henipavirus-specific antibodies at higher rates in females (13/296; 4.4%) than in males (6/234; 2.6%) bats. Concerning the age distribution, we detected antibodies in 2/15 (13.3%) juvenile bats, 4/110 (3.6%) sub-adults, and in 13/406 (3.2%) adults (Table 3).

The multiple sequence alignment (MSA) of host DNA against a reference *Eidolon helvum* genome following the mitochondrial cytochrome b analysis of the serologically reactive bat samples confirmed the phenotypic species identification of all reactive samples as *Eidolon helvum* (Suppl. Fig. 1, Suppl. Fig. 2).

In contrast to the bat transudate samples, all 223 rodent and shrew samples that were available from a total of 300 sampled animals were serologically negative for henipavirus-specific antibodies.

### Molecular analysis

#### RNA analysis for the detection of Henipa-like paramyxovirus in bats and rodents

Although we identified some PCR fragments of the expected length in the bats and rodents, sequencing analysis by Sanger could not confirm that the fragments were related to henipaviruses, which is often the case when degenerate primers are used in a hemi-nested PCR protocol to allow for the detection of multiple virus species, strains and variants. We therefore conclude that we failed to detect henipavirus related RNA in the analyzed pooled samples obtained from bats, rodents and shrews.

## Discussion

### Study design and approach

We decided to target freshly deceased bats for three reasons: firstly, animal welfare considerations question the invasive sampling of wildlife, which brings these animals also in close contact to humans, thereby facilitating zoonotic disease transmissions from animals to humans and vice versa. Secondly, sampling of deceased animals increases the chance of detecting infected animals, even if the infection has not yet been reported to cause clinical disease in the affected animals, but it may well decrease the resilience of the animal to other infections or other external factors. Lastly, bats are grouped under protected species by the Cameroonian regulations.

On the other hand, it needs to be taken into consideration that analyzing tissues of deceased animals bears the risk of degradation of the samples before they can be safely stored at −80°C, even though the carcasses had been collected within a short time after the death of the animal. This approach may however still have had an adverse effect on the detectability of positive samples by serological or molecular analysis, with viral RNA being even less stable than serum antibodies [51, 52].

The testing of rodent and shrew samples was included in this study to shed more light on the possible involvement of these species in the henipavirus epidemiology. For this purpose, sampling was performed in rat infested zones in proximity to human habitations such as homes, restaurants, stores, kitchens, backyards, garbage bins and food markets as well as around isolated swamps, farms and bushlands occasionally visited by humans.

### Serological analysis

One of the key objectives of the study was to develop and evaluate a BMBA using an Indirect ELISA for confirming the detection of henipavirus-specific antibodies in wildlife species in Sub-Saharan Africa such as Cameroon. We applied an assessment scheme where only samples with the results above the cut-off value at two independent measurements and at least one positive result in both assays were considered seropositive. The rationale behind this interpretation scheme is that a sample may yield a false positive result at only one measurement (e.g. due to particles present in the transudate analyzed in this study), as one of the limitations of serological assays is that it can be associated with non-specific reactions [53]. Thus, performing two independent measurements and applying different assays reduces the likelihood of false positive signals. Using this approach, we were able to show a good correlation between the BMBA and the ELISA results, as none of the samples were reactive in only one assay. This confirms the suitability of the BMBA approach for the serological screening of wildlife samples against several antigens in one assay, which is a crucial point when analyzing samples of small animals with limited sample volumes.

Findings of this study added further data regarding the presence of henipaviruses in the Centre Region of Cameroon. It also revealed the first serological evidence of henipavirus infection in seasonal and migratory synanthropic fruit bat (*Eidolon helvum*) from city colonies in the North Region of Cameroon. As shown in Table 3, serological reactivity was revealed mostly in animals that were collected between July and September in the North region or in March and September in the Centre region, which reflects the rainy seasons in these areas. In addition, this is the first serological study performed in Africa to detect antibodies against henipaviruses in bat transudates as opposed to sera which are widely used for serological analysis. Straw-coloured fruit bats (*Eidolon helvum*) are widely distributed in Sub-Saharan Africa, Saudi Arabia and Yemen. They form large and aggregated colonies with thousands of individuals in proximity to human and livestock populations in cities and environs [54]. Additionally, *Eidolon helvum* bats are also hunted as bushmeat in some parts of Africa, including Cameroon. Consequently, there are concerns that potential zoonotic pathogens could spill from bats into human and domesticated animal populations.

Studies reported by others revealed that juvenile and sub-adult bats seem to be more susceptible to infections than adult bats [55]. This indicates that young bats display the highest susceptibility to infection and that this age group may support the circulation of infectious agents within a colony. However, the presence of maternal antibodies cannot be excluded in the juvenile and sub-adult bats. We also detected more positive results in female bats, supporting the finding from other studies [56, 57] where this was correlated to elevated stress during the reproduction phase. Furthermore, we found three clusters of infection in closed colonies, supporting the hypothesis that the agent may be circulating in certain colonies upon introduction through contact of one or several individuals to an infectious source.

In an earlier study, Pernet et al. (2014) reported a higher prevalence of 48% (21/44) in a small and well-curated population of hunted *Eidolon helvum* fruit bats sampled from local hunters in Yaoundé between 2004 and 2007. This was the first study which reported spillover of henipavirus into human populations in Africa [38]. The high prevalence could be attributed to the fact that they tested sera collected immediately after death which may have had higher antibody titers as compared to transudates collected from dead animals as used in our study. Besides, other factors like the use of a different sampling approach, as well as serological assays using different antigens could have contributed to the higher prevalence observed by Pernet et al., (2014). Moreover, the different incidence rates determined in both studies may reflect an oscillation of virus circulation and shedding, with higher levels in 2004–2007 and lower levels in 2018–2020. Lastly, the prevalence shows a high variability between different bat colonies, which we also observed in the colonies sampled in our study.

The BMBA has been used earlier to detect henipavirus-specific antibodies in West African fruit bats (*Eidolon helvum*) from Ghana, revealing seroprevalence rates of 39% and 22% for NiV and HeV respectively [54]. In our study, we did not observe a clear predominance of antibodies raised against either NiV or HeV, while the reactivity towards GhV was generally lower. This may be due to a generally lower reactivity of our recombinant GhV G-Lt protein.

Similarly, HeV G specific antibodies were detected in *Eidolon helvum annobonensis* population at the Annobon Island in the Gulf of Guinea at an incidence rate of 49% in BMBA [58]. In addition, Peel et al., (2013) detected NiV specific antibodies in *Eidolon helvum* bats in Tanzania, East Africa, and reported a seroprevalence of 47.8% (117/245) [59]. Moreso, antibodies against henipaviruses were reported in non-Pteropus bats in China [60] as well as in fruit bats (*Pteropus giganteus*) in India [61].

Findings from this study corroborate with those of Adamu et al., (2022) who reported similar seroprevalence rates in horse and pig populations in Nigeria even though BMBA and ELISA reactive samples were negative in SNT. This also suggests prior exposure of Nigerian livestock to henipaviruses [62].

Interestingly, we did not detect any reactivity of rodents or shrews against HeV G, NiV G and GhV G in serology by BMBA and ELISA, which indicates no circulation of henipaviruses in these species. Regarding the recently detected rodent and shrew associated henipaviruses which are also related to HeV and NiV, nothing is known about their serological cross-reactivity to the other henipaviruses date. Given that the cross-reactivity between CedV and HeV or NiV is lower than that between HeV and NiV [63], the absence of reactive serological results in the rodent and shrew derived transudates does not completely rule out the circulation of rodent or shrew associated henipaviruses in the tested population, but may simply reflect a low cross-reactivity between these viruses.

### Molecular analysis

Another objective of our study was to identify and characterize henipaviruses possibly circulating in fruit bat or rodent and shrew populations from two distinct ecological regions in order to determine any ongoing infection. Our findings revealed that none of the PCR fragments from the pooled samples contained henipavirus related RNA sequences after BLASTn analysis for nucleotide-level analysis and a BLASTx analysis to examine amino acid conservation. Both analyses consistently showed no significant homology with henipaviruses. In conclusion, our approach failed to prove a current circulation of henipaviruses in the analyzed populations, although our serological data show the presence of henipaviruses in the study regions.

In contrast to our results, Drexler et al., (2009), reported the first molecular evidence of henipaviruses in African fruit bats (*Eidolon helvum*) in Ghana with a prevalence of 1.4% (3/215) [16]. Moreover, the whole genome of Ghana virus (GhV) was successfully sequenced in a follow-up study [64]. In addition, Weiss et al., (2012), reported henipavirus detection in *Eidolon helvum* fruit bats from the Republic of Congo at an incidence rate of 15/339 (4.4%) [65]. More recently, a novel bat-derived henipa-like paramyxovirus designated Angavokely virus (AngV) was detected and characterized from fruit bats (*Eidolon dupreanum*) using the metagenomics Next Generation Sequencing (mNGS) approach [17] thereby increasing the number of African henipavirus species and its geographic expansion and host species.

The difference in results observed between our study as opposed to others could be attributed to the ecology, season and geographic location, physiological state of the bats and the virus shedding pattern [55]. As discussed for the transudate samples, sampling of deceased animals may have interfered with the integrity of the viral nucleic acid, but nevertheless this approach should have considerably increased the probability to detect infected animals. The fact that the amplification of the cytochrome b gene for species identification of the positive animals was unproblematic argues against an extensive RNA degradation in the samples.

In contrast to our findings where we detected no henipa-like paramyxovirus RNA sequences in rodents and shrews, a few findings on rodent paramyxoviruses have recently been documented in Africa. Onyuok et al., (2019) detected and characterized Paramyxoviruses in wild and synanthropic rodents and shrews in Kenya with a prevalence of 7/617 (1.1%) [21]. Similarly, in another study to characterize paramyxoviruses molecularly in wild rodents and shrews in Zambia, paramxoviruses were detected at an incident rate of 96/462 (20.8%). Phylogenetic analysis revealed that these viruses were novel paramyxoviruses and could be classified as morbilliviruses and henipaviruses as well as previously identified rodent paramyxoviruses [20]. However, the vast majority of positive results in these studies were obtained for wild rodent species, while only one out of 54 analyzed *Rattus rattus* sample was found positive for henipaviruses [20]. In China, the rodent species *Rattus flavipectus* was shown to be the reservoir hosts for the Mojiang virus (MojV), the first rodent-borne henipavirus [19]. Recently, two shrew-derived henipavirus species respectively designated Gamak virus (GakV) and Daeryong virus (DarV) were discovered and characterized from shrews in the Republic of Korea by whole genome sequencing involving mNGS, Illumina MiSeq and HiSeq approaches [26]. However, our results corroborate with those of Berto et al., (2018), who reported the absence of paramyxovirus RNA sequences in Vietnamese rodents [66].

Nevertheless, future in-depth studies involving whole genome NGS and metagenomics approaches and phylogenetic analysis could be adopted instead of targeted Sanger sequencing. These advanced techniques, contrary to the Sanger sequencing will provide more substantial information on the origin, genetic diversity and circulating species of bat-and-rodent-borne potentially zoonotic paramyxoviruses and other high public impact pathogens.

## Conclusions

In this study, we screened 531 bat transudates using a BMBA approach using an indirect ELISA for confirmation. Using this approach we not only confirmed the presence of henipavirus specific antibodies in seasonal and synanthropic *Eidolon helvum* fruit bats roosting and breeding in proximity to humans in Yaoundé located in the Centre region of Cameroon. Although the detected incidence was lower than that reported in earlier studies, which may be partly due to our study design, we were for the first time able to provide serological proof of henipa-like paramyxoviruses in fruit bat populations in the North Region of Cameroon. However, we failed to confirm the circulation of henipavirus-related RNA sequences in the sampled bat, rodent and shrew populations in the Centre and North Regions of Cameroon at the time of sampling.

The data from this study can serve as basis for a follow-up assessment of the risk of emergence of potentially zoonotic pathogens from fruit bats in Cameroon. Surveillance studies involving a One Health approach at the human-livestock-wildlife interface may provide additional information on possible spillover events caused by henipavirus species and other high impact viruses. This will allow targeted control measures and minimize public health risks caused by emerging and zoonotic diseases.

## Methods

### Ethical and administrative approvals

Approvals for bat and rodent sampling were obtained from the Cameroon Ministry of Research and Scientific Innovation – MINRESI (N^o^000000311/MINRESI/B00/C00/C10/C12) and the Ministry of Wildlife and Forestry – MINFOF (N^o^0183 PRS/MINFOF/SG/DFAP/SDVEF/SC/MC), and were renewed yearly during the sampling period from March 2018 to June 2020.

### Sample size calculation

The sample size was calculated based on the unknown population size of N = ∞ (> 10000) and an estimated prevalence of 1%. Thus, a sample size of *n* = 299 was required to detect at least one positive animal with a statistical power of 95% by applying the derivative of probability function of hypergeometric distribution [67]. Consequently, a minimum sample size of 300 animals/ region for each species was required. A total of 600 bats and 600 rodents were collected during this study.

### Bat and rodent sampling

Sampling was performed in two different ecological regions of Cameroon; the Centre Region (Savanna transition and Forest zone, characterized by two rainy and dry seasons) and the North Region (Sudano-Sahelian zone, characterized by one long rainy and a dry season). Selection of bat sampling sites was based on available data and field observations of fruit bats in the various regions under investigation.

**Fig. 2:**
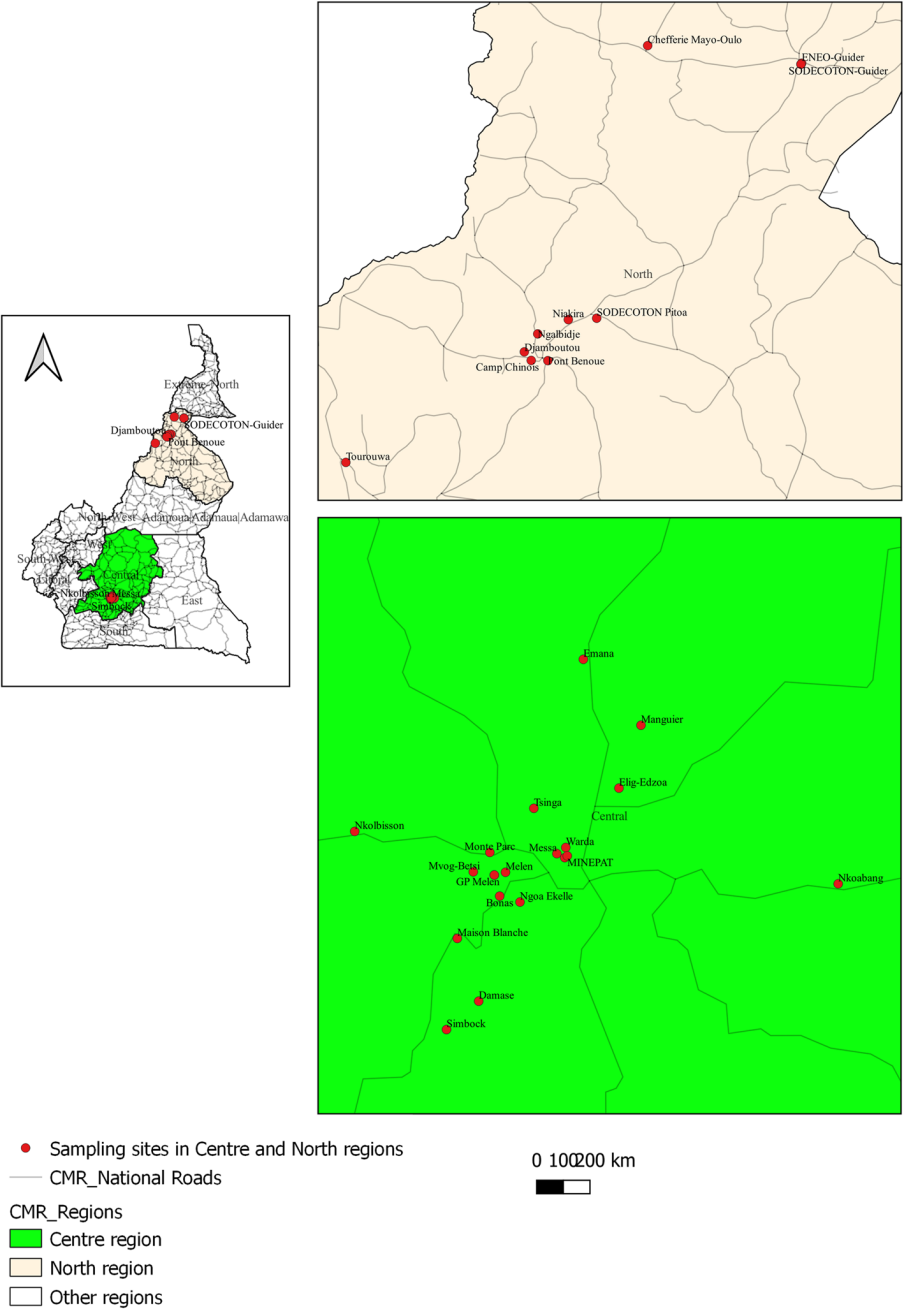
Bat and rodent sampling sites in the Centre and North Regions of Cameroon

In the Centre Region, bat samples were collected at known fruit bat roost sites in Yaounde such as Cascades de Mfoundi, Warda - Bois Sainte, Anastasie, GP Melen, Messa and MINEPAT. Whereas in the North Region (Garoua), sampling was done at Sodecoton-Guider, ENEO-Guider, Chefférie Mayo-Oulo and Sodecoton-Pitoa. Details of sampling sites are illustrated in Fig. 2 and Suppl. Table 1. Bat colonies were visited at least three times weekly from March 2018 to April 2020. Freshly found dead bats were collected under their roost sites and were transported in individual plastic zip-lock bags to the *Laboratoire National Vetérinaire* (LANAVET) Annexe de Yaounde and the LANAVET Garoua under refrigerated conditions for further processing. Our rationale to collect freshly deceased bats was that these animals will have an increased likelihood of carrying disease agents of any kind, including the ones that this specific study was targeting. Since on the other hand, autolytic processes will start soon upon death under the given climatic conditions, the carcasses were collected early in the morning and immediately transferred to the laboratory under cooled conditions.

In the Centre Region, rodents were sampled around the following neighborhoods; Bonas, Emana, Mvog-Betsi, Nkolbisson, Monté Parc, Maison Blanche, Melen, Ngoa-Ekelle, Elig-Edzoa, Simbock, Manguier, Damase and Tsinga. In the North Region, sampling was done at Sodecoton-Guider, Djamboutou, Ngalbidje, Niakira, Pont Benoue, Camp Chinois and Tourouwa (Fig. 2 and Suppl. Table 2). Baited traps were set every morning and evening at rat infested areas such as homes, restaurants, stores, kitchens, backyards, swamps, garbage bins, food markets, bushlands and farms. Traps were checked hourly during the day while traps set late in the evening, were verified the next day, early in the morning. Sampling was performed from March 2018 to July 2020. Rodents and shrews were collected depending on the freshness of the dead rat carcasses. Carcasses were then transported under refrigerated conditions to the necropsy unit at LANAVET for further processing. During the necropsies, staff were wearing Personal Protective Equipment (PPE) consisting of a Tyvek coverall, double layer of nitril gloves, N95 respirator, safety goggles, shoe covers and an apron. All necropsies were performed in a class II Biological Safety Cabinet (BSC) to minimize the risk of exposure to potential zoonotic pathogens.

### Sample processing

Samples were registered with a unique identification number and information about the collection sites included GPS coordinates, species, gender, age class (based on the presence of secondary characteristics), weight and specimen (Table 1; Suppl. Table 4). Animals were initially phenotypically identified with photographic identification guides based on morphometric parameters such as color, forearm length, weight, tail length, head-body length and presence of primary reproductive organs (penis, testes, vaginal orifice and mammary glands). Necropsy of bats and rodents were conducted in a BSC II following guidelines from the Predict One Health Consortium 2016 [68, 69]. Transudates, liver, intestines, lungs, brain, spleen and kidneys were collected from each bat. For rodents and shrews, only transudate, liver, lungs, brain and spleen were collected. Samples for serological analysis were inactivated by adding Sodium Dodecyl Sulphate (SDS) to a final concentration of 0.5% and incubation at 56°C for 1 h. This procedure did not interfere with the reactivity of the serum samples against the antigens used in this study (Table 2). To test this, we used three negative sera (pig, rabbit, bat) and six sera of pigs, rabbits or bats that were immunized with one of the henipavirus antigens used in our study. These sera were then subjected to a 1 h incubation at 56°C with or without the addition of 0.5% SDS, and analyzed by BMBA and ELISA. Samples for molecular analysis were placed in Trizol to inactivate infectious agents and stabilize the RNA. All samples were stored at −80^o^C prior to laboratory analysis.

### Serological analysis for the detection of henipavirus-specific antibodies

A total of 754 transudate specimens (531 from bats and 223 from rodents) were collected under sterile conditions preventing cross-contamination during necropsy at the LANAVET in Cameroon and serologically analyzed at the Friedrich-Loeffler-Institute (FLI), Insel Riems in Germany. The small body size of some of the specimens impeded the collection of transudate. Samples were initially screened for antibodies against henipaviruses using a BMBA based on a 10 bead (microsphere) panel for which individually labelled magnetic beads (microspheres) were coated with recombinant Hendra virus G glycoprotein (HeV G-Lt), Nipah virus G glycoprotein (NiV G-Lt), Ghana virus G glycoprotein (GhV G-Lt) expressed in *L. tarentolae* (Lt) following the protocol published in [46], and other antigens that were not relevant for this study (Rift Valley Fever Virus nucleoprotein, Hepatitis E Virus p239, Lagos bat Virus glycoprotein, Usutu Virus, West Nile Virus and Tick-borne Encephalitis Virus NS1 proteins). This assay was repeated in an independent approach using only HeV G-Lt and NiV G-Lt. Samples were further analyzed to confirm the results obtained in the BMBA using an in-house indirect ELISA specific for HeV G-Lt and NiV G-Lt based on a published protocol [47] for all samples, and for all reactive samples in another ELISA experiment. In addition, all reactive samples were subjected to both BMBA and ELISA using commercially available (c) HeV G-c and NiV G-c glycoproteins (AcroBiosystems, Newark Delaware, USA). Thus altogether, each sample which was recorded as reactive during the first BMBA analysis was repeated in at least 5 additional assays (another round of BMBA analyzing all samples using recombinant proteins expressed in *L. tarentolae*, two independent ELISA analyses using recombinant proteins expressed in *L. tarentolae*, and finally one BMBA and one ELISA using the commercially available proteins). Only samples giving reactive results in at least four out of these six approaches were considered positive in our serological analysis.

Serum samples from bats and rabbits immunized with the aforementioned recombinant antigens were used as positive controls, and sera collected from the same animals prior to immunization served as negative controls.

The ELISA has previously been thoroughly validated for the screening of pig and horse samples [47, 49]. As shown in Table 2, both the BMBA and ELISA easily detect positive serum samples from in-house immunized pigs, rabbits and fruit bats.

### Bead-based multiplexed binding assay (BMBA)

Magnetic beads were coated with the respective recombinant proteins (HeV G-Lt, NiV G-Lt, GhV G-Lt, HeV G-c, NiV-c) using the Bio-Plex Amine Coupling Kit (BioRad, Munich, Germany). The coated magnetic beads were vortexed for 30s at medium speed, and sonicated by bath sonication for 15s at 70% power, and vortexed once more for 30s at medium speed. A volume of 0.5µL of each coated bead in 50µL bead buffer were added to appropriate wells of a black flat bottom plate (Greiner, Frickenhausen, Germany). The bead buffer was removed by placing the plate on a magnetic plate holder (BioRad, Munich, Germany) for 2 min, followed by careful aspiration of the buffer. Next, 50µL of bat and rodent transudates or control sera diluted at 1:100 in PBST (Gibco 1X Dulbecco’s Phosphate Buffered Saline – DPBS plus 0.05% Tween 20) were added to the wells. The plate was covered and placed on a plate shaker at room temperature (RT) at 850 rpm for 1 h. The plate was washed 3 times with 100µL/well PBST before 50µL of a mouse anti-human IgG (BioLegend, San Diego, USA; RRID: AB_2650624) showing a broad reactivity to a variety of mammalian species, diluted at 1:200 PBST was added per well and the plate was incubated at 850 rpm for 1 h at RT. The plate was washed 3 times with 100µL/well PBST. Then 50µL of Alexa fluor 532 goat anti-mouse IgG (Life Technologies, Darmstadt, Germany; RRID: AB_2534070) diluted at 1:100 PBST was added per well and the plate was incubated at 850 rpm for 1 h at RT. The plate was again washed 3 times with 100µL/well PBST before 125µL of Sheath buffer was added to each well and the plate was placed on a shaker at 850 rpm for 2 min at RT. Finally, the Median Fluorescence Intensity (MFI) was measured using the Bio-Rad Bio-Plex 200 system. Based on published cut-off values in similar approaches [57], we first set a provisional BMBA cut-off value of MFI 400 for all antigens, and then calculated the specific cut-off values for each antigen expressed as mean value plus 3 times the standard deviation (SD) of the sera that were then considered negative.

All samples with MFI values above the specific cut-off value were re-analyzed in the other assays on separate dates. To confirm the results obtained with the *in-house* recombinant proteins expressed in *L. tarentolae*, we repeated the assay using commercially available HeV G and NiV G proteins (AcroBiosystems, Newark Delaware, USA).

### Indirect ELISA

Initially BMBA reactive samples were further analyzed using an in-house Indirect ELISA. Succinctly, 96 wells plates (Nunc Medisorp) were coated over night at 4°C with 100µL/ well of 1µg/mL recombinant HeV G-Lt, NiV G-Lt, HeV G-c or NiV G-c proteins in Phosphate Buffered Saline (PBS). The plates were washed one time using 150µL/ well of PBST, followed by blocking with 100µL/well 5% skimmed milk in PBS and then incubated at 37^o^C for 1 hour. The plates were washed 3 times with 150µL/well PBST. Then 100µl/well of the BMBA-reactive bat transudates diluted at 1:100 in 2.5% skimmed milk in PBS were added and plates were incubated at 37^o^C for 1 hour. The plates were washed 3 times with 150µl/well PBST and 100µl of a purified recombinant Peroxidase conjugated Protein A/G (Thermo Fisher Scientific, Schwerte, Germany) in a dilution of 1:30,000 was added per well and the plate was incubated at 37^o^C for 1 hour. Plates were washed 3 times with 150µl/well PBST, then 100µl/well of 3,3’,5,5’-Tetramethylbenzidene (TMB) were added and the plate was incubated in the dark at RT for 10 min. The reaction was stopped by adding 100 µl/well of 1 M sulfuric acid (H_2_SO_4_), and the optical density (OD) was measured at 450 nm using an ELISA plate reader spectrophotometer (Tecan, Trading AG, Männedorf, Switzerland).

### Molecular analysis

#### Viral RNA extraction

The Trizol-treated samples from bats (lungs, brain, transudate and liver) and rodents as well as shrews (brain, liver and transudate) were homogenized and pooled per animal, that is four specimens per bat for each pool, and three specimens per rodent or shrew for each pool. Bat, rodent and shrew samples were pooled separately. Thus, a total of 1200 sample pools (600 bats and 600 rodents / shrews) were generated and RNA was extracted using RNeasy mini kit (Qiagen, Hilden, Germany) according to the manufacturer’s instructions. Cell culture supernatant infected with Bovine Parainfluenza Virus 3 in AVL buffer served as positive control for the hemi-nested RT-PCR and viral RNA was extracted using QIAamp Viral RNA mini kit (Qiagen) according to the manufacturer’s instructions. After elution, all samples were preserved at −80^o^C until PCR analysis.

#### Reverse transcription polymerase chain reaction (RT-PCR)

A broad spectrum hemi-nested RT-PCR assay with degenerate primers (RES-MOR-HEN-F1, RES-MOR-HEN-F2, RES-MOR-HEN-R) targeting a highly conserved region of the Large Polymerase gene (L-gene) of *Respiro-Morbilli-*and-*Henipa-related paramyxoviruses* was applied [70]. The first PCR amplification was done with the qScript XLT 1-Step RT-PCR Kit (Quantabio, Beverly, MA, USA) while the second amplification was performed using Pwo DNA Polymerase (Merck, Taufkirchen, Germany). Amplicons were resolved by electrophoresis on a 2% (w/v) agarose gel stained with Gel-Red and the expected band size was confirmed at 495 bp. The Parainfluenza Virus 3 served as positive control. We have applied this procedure successfully in a previous screening study of German bats for the presence of paramyxovirus related RNA [71]. All samples from a pool revealing a specific band of the expected length were re-analyzed individually.

#### Sequencing of PCR products of the expected size

To further characterize the PCR fragments, individual samples were shipped to INQABA’s commercial sequencing service in Pretoria, South Africa. Prior to the sequencing, the PCR products were reamplified using the NEB One Taq 2X Master Mix with Standard Buffer (New England Biolabs, Ipswich, USA) according to the manufacturer’s instructions. The amplicons obtained were electrophoresed and integrity of the PCR amplicons was visualized on a 1% agarose gel (CSL-AG500, Cleaver Scientific Ltd, Rugby, UK) stained with EZ-vision^®^ Bluelight DNA Dye (Avantor, Radnor, USA). PCR products were excised and cleaned using Exonuclease I and Shrimp Alkaline Phosphatase (ExoSAP-IT) kit (New England Biolabs, Ipswich, USA) according to the manufacturer’s instructions. PCR fragments were labelled using the Nimagen BrilliantDye™ Terminator Cycle Sequencing Kit V3.1, (Thermo Fisher Scientific, Waltham, USA) according to manufacturer’s instructions and the labelled products were cleaned with the ZR-96 DNA Sequencing Clean-up Kit (Zymo Research, Irvine, USA) according to the manufacturer’s instructions. Finally, the cleaned products were injected in an Applied Biosystems ABI 3730XL Genetic Analyser with a 50 cm array and sequencing was done in both forward and reverse directions.

#### Species identification

We confirmed the species for all bats that were suspected or confirmed to be positive for henipavirus specific antibodies by mitochondrial cytochrome b gene analysis as described in Stoek et al., (2022) with minor modifications. Briefly, the extracted DNA was analyzed in a PCR protocol for mitochondrial cytochrome b gene of different species [72] using the QuantiTect Multiplex PCR NoROX Kit (Qiagen, Hilden, Germany). PCR products were verified by gel electrophoresis in a 1.5% agarose gel. Gels were stained with ethidium bromide for evaluation. PCR products of samples showing a specific band (464 bp) in the gel were sent for sequencing [73].

### Data processing and analysis

Microsoft Excel version 2016 and the IBM^®^ Statistical Package for Social Sciences Software (SPSS) version 20 was used to perform descriptive statistics (mean, standard deviation and proportions (percentages)), The Geographic Information System (Q-GIS) version 3.16 was used to design maps of study sites based on GPS coordinates collected at different bat and rodent sampling sites. The GPS coordinates were imported into Q-GIS and shapefiles of Cameroon with administrative divisions were downloaded online and imported accordingly.

Raw chromatogram data were processed with Sequence Scanner version 1.0 (Applied Biosystems), BioEdit version 7.2.5 and Seqman Ultra – DNASTAR Lasergene version 17.3. For the latter, trimming was done at a minimum match percentage of 80%, applying both quality and vector trimming prior to assembly. Additional data processing to obtain frameshifts and indels accuracy in consensus sequences was performed with Expasy (https://web.expasy.org/translate/). Subsequently, a reference-based alignment to find homologues was done using nucleotide BLAST (BLASTn). Multiple sequence alignment (MSA) was performed using ClustalW [74].

## Supplementary Information

Supplementary Material 1.

## Data Availability

The datasets used and/or analyzed during the current study are available from the corresponding authors on request.
