# Supplementary material for: Serological and molecular analysis of henipavirus infections in synanthropic fruit bat and rodent populations in the Centre and North regions of Cameroon (2018–2020)
Source: BMC Vet Res. 2025 Feb 24;21:93. doi: 10.1186/s12917-025-04530-4 (PMC11849310; doi:10.1186/s12917-025-04530-4)
Supplement: Supplementary file 1 — Supplementary Material 1. [file 12917_2025_4530_MOESM1_ESM.docx]

**Supplementary Material to:**

**Serological and Molecular Analysis of Henipavirus Infections in Synanthropic Fruit Bat and Rodent Populations in the Centre and North Regions of Cameroon (2018-2020)**

Cyrille Mbanwi Mbu’u, Pierre Gontao, Abel Wade, Maren Penning, Balal Sadeghi, Aristid Ekollo Mbange, Matthew LeBreton, Sylvain Leroy Sado Kamdem, Franziska Stoek, Martin Hermann Groschup, Wilfred Fon Mbacham, Anne Balkema-Buschmann


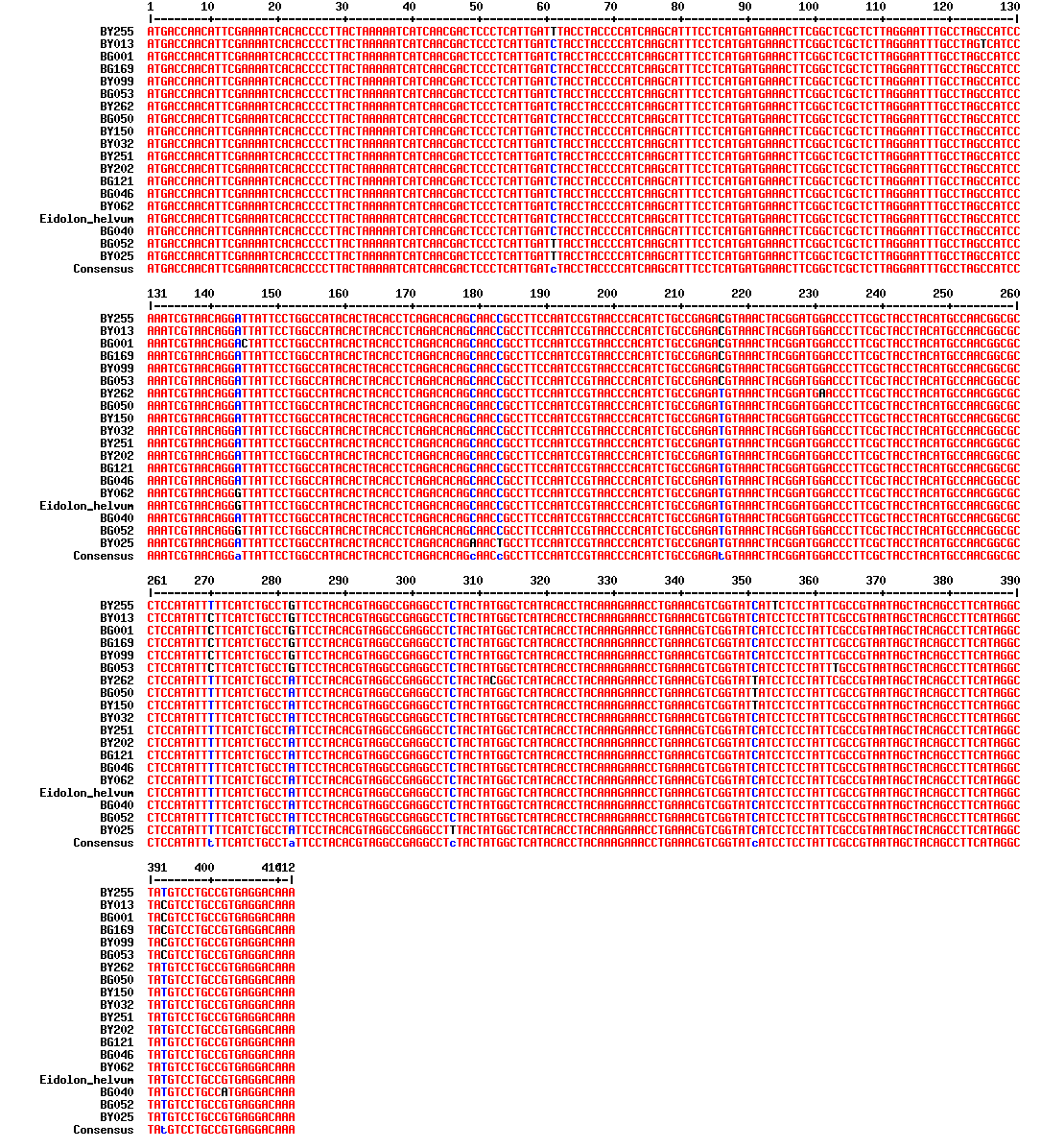
Supplementary Figure 1: Multiple sequence alignment of host DNA in reactive samples to reference *Eidolon helvum* sequence. Red indicates conserved residues across all sequences, blue color indicates residues with a low consensus, and black indicates residues with a neutral consensus.

>BY255

ATGACCAACATTCGAAAATCACACCCCTTACTAAAAATCATCAACGACTCCCTCATTGATTTACCTACCCCATCAAGCATTTCCTCATGATGAAACTTCGGCTCGCTCTTAGGAATTTGCCTAGCCATCCAAATCGTAACAGGATTATTCCTGGCCATACACTACACCTCAGACACAGCAACCGCCTTCCAATCCGTAACCCACATCTGCCGAGACGTAAACTACGGATGGACCCTTCGCTACCTACATGCCAACGGCGCCTCCATATTTTTCATCTGCCTGTTCCTACACGTAGGCCGAGGCCTCTACTATGGCTCATACACCTACAAAGAAACCTGAAACGTCGGTATCATTCTCCTATTCGCCGTAATAGCTACAGCCTTCATAGGCTATGTCCTGCCGTGAGGACAAA

>BY013

ATGACCAACATTCGAAAATCACACCCCTTACTAAAAATCATCAACGACTCCCTCATTGATCTACCTACCCCATCAAGCATTTCCTCATGATGAAACTTCGGCTCGCTCTTAGGAATTTGCCTAGTCATCCAAATCGTAACAGGATTATTCCTGGCCATACACTACACCTCAGACACAGCAACCGCCTTCCAATCCGTAACCCACATCTGCCGAGACGTAAACTACGGATGGACCCTTCGCTACCTACATGCCAACGGCGCCTCCATATTCTTCATCTGCCTGTTCCTACACGTAGGCCGAGGCCTCTACTATGGCTCATACACCTACAAAGAAACCTGAAACGTCGGTATCATCCTCCTATTCGCCGTAATAGCTACAGCCTTCATAGGCTACGTCCTGCCGTGAGGACAAA

>BG053

ATGACCAACATTCGAAAATCACACCCCTTACTAAAAATCATCAACGACTCCCTCATTGATCTACCTACCCCATCAAGCATTTCCTCATGATGAAACTTCGGCTCGCTCTTAGGAATTTGCCTAGCCATCCAAATCGTAACAGGATTATTCCTGGCCATACACTACACCTCAGACACAGCAACCGCCTTCCAATCCGTAACCCACATCTGCCGAGACGTAAACTACGGATGGACCCTTCGCTACCTACATGCCAACGGCGCCTCCATATTCTTCATCTGCCTGTTCCTACACGTAGGCCGAGGCCTCTACTATGGCTCATACACCTACAAAGAAACCTGAAACGTCGGTATCATCCTCCTATTTGCCGTAATAGCTACAGCCTTCATAGGCTACGTCCTGCCGTGAGGACAAA

>BG001

ATGACCAACATTCGAAAATCACACCCCTTACTAAAAATCATCAACGACTCCCTCATTGATCTACCTACCCCATCAAGCATTTCCTCATGATGAAACTTCGGCTCGCTCTTAGGAATTTGCCTAGCCATCCAAATCGTAACAGGACTATTCCTGGCCATACACTACACCTCAGACACAGCAACCGCCTTCCAATCCGTAACCCACATCTGCCGAGACGTAAACTACGGATGGACCCTTCGCTACCTACATGCCAACGGCGCCTCCATATTCTTCATCTGCCTGTTCCTACACGTAGGCCGAGGCCTCTACTATGGCTCATACACCTACAAAGAAACCTGAAACGTCGGTATCATCCTCCTATTCGCCGTAATAGCTACAGCCTTCATAGGCTACGTCCTGCCGTGAGGACAAA

>BG169

ATGACCAACATTCGAAAATCACACCCCTTACTAAAAATCATCAACGACTCCCTCATTGATCTACCTACCCCATCAAGCATTTCCTCATGATGAAACTTCGGCTCGCTCTTAGGAATTTGCCTAGCCATCCAAATCGTAACAGGATTATTCCTGGCCATACACTACACCTCAGACACAGCAACCGCCTTCCAATCCGTAACCCACATCTGCCGAGACGTAAACTACGGATGGACCCTTCGCTACCTACATGCCAACGGCGCCTCCATATTCTTCATCTGCCTGTTCCTACACGTAGGCCGAGGCCTCTACTATGGCTCATACACCTACAAAGAAACCTGAAACGTCGGTATCATCCTCCTATTCGCCGTAATAGCTACAGCCTTCATAGGCTACGTCCTGCCGTGAGGACAAA

>BY099

ATGACCAACATTCGAAAATCACACCCCTTACTAAAAATCATCAACGACTCCCTCATTGATCTACCTACCCCATCAAGCATTTCCTCATGATGAAACTTCGGCTCGCTCTTAGGAATTTGCCTAGCCATCCAAATCGTAACAGGATTATTCCTGGCCATACACTACACCTCAGACACAGCAACCGCCTTCCAATCCGTAACCCACATCTGCCGAGACGTAAACTACGGATGGACCCTTCGCTACCTACATGCCAACGGCGCCTCCATATTCTTCATCTGCCTGTTCCTACACGTAGGCCGAGGCCTCTACTATGGCTCATACACCTACAAAGAAACCTGAAACGTCGGTATCATCCTCCTATTCGCCGTAATAGCTACAGCCTTCATAGGCTACGTCCTGCCGTGAGGACAAA

>BY025

ATGACCAACATTCGAAAATCACACCCCTTACTAAAAATCATCAACGACTCCCTCATTGATTTACCTACCCCATCAAGCATTTCCTCATGATGAAACTTCGGCTCGCTCTTAGGAATTTGCCTAGCCATCCAAATCGTAACAGGATTATTCCTGGCCATACACTACACCTCAGACACAGAAACTGCCTTCCAATCCGTAACCCACATCTGCCGAGATGTAAACTACGGATGGACCCTTCGCTACCTACATGCCAACGGCGCCTCCATATTTTTCATCTGCCTATTCCTACACGTAGGCCGAGGCCTTTACTATGGCTCATACACCTACAAAGAAACCTGAAACGTCGGTATCATCCTCCTATTCGCCGTAATAGCTACAGCCTTCATAGGCTATGTCCTGCCGTGAGGACAAA

>BY262

ATGACCAACATTCGAAAATCACACCCCTTACTAAAAATCATCAACGACTCCCTCATTGATCTACCTACCCCATCAAGCATTTCCTCATGATGAAACTTCGGCTCGCTCTTAGGAATTTGCCTAGCCATCCAAATCGTAACAGGATTATTCCTGGCCATACACTACACCTCAGACACAGCAACCGCCTTCCAATCCGTAACCCACATCTGCCGAGATGTAAACTACGGATGAACCCTTCGCTACCTACATGCCAACGGCGCCTCCATATTTTTCATCTGCCTATTCCTACACGTAGGCCGAGGCCTCTACTACGGCTCATACACCTACAAAGAAACCTGAAACGTCGGTATTATCCTCCTATTCGCCGTAATAGCTACAGCCTTCATAGGCTATGTCCTGCCGTGAGGACAAA

>BG050

ATGACCAACATTCGAAAATCACACCCCTTACTAAAAATCATCAACGACTCCCTCATTGATCTACCTACCCCATCAAGCATTTCCTCATGATGAAACTTCGGCTCGCTCTTAGGAATTTGCCTAGCCATCCAAATCGTAACAGGATTATTCCTGGCCATACACTACACCTCAGACACAGCAACCGCCTTCCAATCCGTAACCCACATCTGCCGAGATGTAAACTACGGATGGACCCTTCGCTACCTACATGCCAACGGCGCCTCCATATTTTTCATCTGCCTATTCCTACACGTAGGCCGAGGCCTCTACTATGGCTCATACACCTACAAAGAAACCTGAAACGTCGGTATTATCCTCCTATTCGCCGTAATAGCTACAGCCTTCATAGGCTATGTCCTGCCGTGAGGACAAA

>BY150

ATGACCAACATTCGAAAATCACACCCCTTACTAAAAATCATCAACGACTCCCTCATTGATCTACCTACCCCATCAAGCATTTCCTCATGATGAAACTTCGGCTCGCTCTTAGGAATTTGCCTAGCCATCCAAATCGTAACAGGATTATTCCTGGCCATACACTACACCTCAGACACAGCAACCGCCTTCCAATCCGTAACCCACATCTGCCGAGATGTAAACTACGGATGGACCCTTCGCTACCTACATGCCAACGGCGCCTCCATATTTTTCATCTGCCTATTCCTACACGTAGGCCGAGGCCTCTACTATGGCTCATACACCTACAAAGAAACCTGAAACGTCGGTATTATCCTCCTATTCGCCGTAATAGCTACAGCCTTCATAGGCTATGTCCTGCCGTGAGGACAAA

>BY032

ATGACCAACATTCGAAAATCACACCCCTTACTAAAAATCATCAACGACTCCCTCATTGATCTACCTACCCCATCAAGCATTTCCTCATGATGAAACTTCGGCTCGCTCTTAGGAATTTGCCTAGCCATCCAAATCGTAACAGGATTATTCCTGGCCATACACTACACCTCAGACACAGCAACCGCCTTCCAATCCGTAACCCACATCTGCCGAGATGTAAACTACGGATGGACCCTTCGCTACCTACATGCCAACGGCGCCTCCATATTTTTCATCTGCCTATTCCTACACGTAGGCCGAGGCCTCTACTATGGCTCATACACCTACAAAGAAACCTGAAACGTCGGTATCATCCTCCTATTCGCCGTAATAGCTACAGCCTTCATAGGCTATGTCCTGCCGTGAGGACAAA

>BG046

ATGACCAACATTCGAAAATCACACCCCTTACTAAAAATCATCAACGACTCCCTCATTGATCTACCTACCCCATCAAGCATTTCCTCATGATGAAACTTCGGCTCGCTCTTAGGAATTTGCCTAGCCATCCAAATCGTAACAGGATTATTCCTGGCCATACACTACACCTCAGACACAGCAACCGCCTTCCAATCCGTAACCCACATCTGCCGAGATGTAAACTACGGATGGACCCTTCGCTACCTACATGCCAACGGCGCCTCCATATTTTTCATCTGCCTATTCCTACACGTAGGCCGAGGCCTCTACTATGGCTCATACACCTACAAAGAAACCTGAAACGTCGGTATCATCCTCCTATTCGCCGTAATAGCTACAGCCTTCATAGGCTATGTCCTGCCGTGAGGACAAA

>BG121

ATGACCAACATTCGAAAATCACACCCCTTACTAAAAATCATCAACGACTCCCTCATTGATCTACCTACCCCATCAAGCATTTCCTCATGATGAAACTTCGGCTCGCTCTTAGGAATTTGCCTAGCCATCCAAATCGTAACAGGATTATTCCTGGCCATACACTACACCTCAGACACAGCAACCGCCTTCCAATCCGTAACCCACATCTGCCGAGATGTAAACTACGGATGGACCCTTCGCTACCTACATGCCAACGGCGCCTCCATATTTTTCATCTGCCTATTCCTACACGTAGGCCGAGGCCTCTACTATGGCTCATACACCTACAAAGAAACCTGAAACGTCGGTATCATCCTCCTATTCGCCGTAATAGCTACAGCCTTCATAGGCTATGTCCTGCCGTGAGGACAAA

>BY202

ATGACCAACATTCGAAAATCACACCCCTTACTAAAAATCATCAACGACTCCCTCATTGATCTACCTACCCCATCAAGCATTTCCTCATGATGAAACTTCGGCTCGCTCTTAGGAATTTGCCTAGCCATCCAAATCGTAACAGGATTATTCCTGGCCATACACTACACCTCAGACACAGCAACCGCCTTCCAATCCGTAACCCACATCTGCCGAGATGTAAACTACGGATGGACCCTTCGCTACCTACATGCCAACGGCGCCTCCATATTTTTCATCTGCCTATTCCTACACGTAGGCCGAGGCCTCTACTATGGCTCATACACCTACAAAGAAACCTGAAACGTCGGTATCATCCTCCTATTCGCCGTAATAGCTACAGCCTTCATAGGCTATGTCCTGCCGTGAGGACAAA

>BY251

ATGACCAACATTCGAAAATCACACCCCTTACTAAAAATCATCAACGACTCCCTCATTGATCTACCTACCCCATCAAGCATTTCCTCATGATGAAACTTCGGCTCGCTCTTAGGAATTTGCCTAGCCATCCAAATCGTAACAGGATTATTCCTGGCCATACACTACACCTCAGACACAGCAACCGCCTTCCAATCCGTAACCCACATCTGCCGAGATGTAAACTACGGATGGACCCTTCGCTACCTACATGCCAACGGCGCCTCCATATTTTTCATCTGCCTATTCCTACACGTAGGCCGAGGCCTCTACTATGGCTCATACACCTACAAAGAAACCTGAAACGTCGGTATCATCCTCCTATTCGCCGTAATAGCTACAGCCTTCATAGGCTATGTCCTGCCGTGAGGACAAA

>BG040

ATGACCAACATTCGAAAATCACACCCCTTACTAAAAATCATCAACGACTCCCTCATTGATCTACCTACCCCATCAAGCATTTCCTCATGATGAAACTTCGGCTCGCTCTTAGGAATTTGCCTAGCCATCCAAATCGTAACAGGATTATTCCTGGCCATACACTACACCTCAGACACAGCAACCGCCTTCCAATCCGTAACCCACATCTGCCGAGATGTAAACTACGGATGGACCCTTCGCTACCTACATGCCAACGGCGCCTCCATATTTTTCATCTGCCTATTCCTACACGTAGGCCGAGGCCTCTACTATGGCTCATACACCTACAAAGAAACCTGAAACGTCGGTATCATCCTCCTATTCGCCGTAATAGCTACAGCCTTCATAGGCTATGTCCTGCCATGAGGACAAA

>BG052

ATGACCAACATTCGAAAATCACACCCCTTACTAAAAATCATCAACGACTCCCTCATTGATTTACCTACCCCATCAAGCATTTCCTCATGATGAAACTTCGGCTCGCTCTTAGGAATTTGCCTAGCCATCCAAATCGTAACAGGGTTATTCCTGGCCATACACTACACCTCAGACACAGCAACCGCCTTCCAATCCGTAACCCACATCTGCCGAGATGTAAACTACGGATGGACCCTTCGCTACCTACATGCCAACGGCGCCTCCATATTTTTCATCTGCCTATTCCTACACGTAGGCCGAGGCCTCTACTATGGCTCATACACCTACAAAGAAACCTGAAACGTCGGTATCATCCTCCTATTCGCCGTAATAGCTACAGCCTTCATAGGCTATGTCCTGCCGTGAGGACAAA

>BY062

ATGACCAACATTCGAAAATCACACCCCTTACTAAAAATCATCAACGACTCCCTCATTGATCTACCTACCCCATCAAGCATTTCCTCATGATGAAACTTCGGCTCGCTCTTAGGAATTTGCCTAGCCATCCAAATCGTAACAGGGTTATTCCTGGCCATACACTACACCTCAGACACAGCAACCGCCTTCCAATCCGTAACCCACATCTGCCGAGATGTAAACTACGGATGGACCCTTCGCTACCTACATGCCAACGGCGCCTCCATATTTTTCATCTGCCTATTCCTACACGTAGGCCGAGGCCTCTACTATGGCTCATACACCTACAAAGAAACCTGAAACGTCGGTATCATCCTCCTATTCGCCGTAATAGCTACAGCCTTCATAGGCTATGTCCTGCCGTGAGGACAAA

Supplementary Figure 2: sequence data of PCR products of the heminested PCR targeting a highly conserved region of the Large Polymerase gene (L-gene) of Respiro-Morbilli-and-Henipa-related paramyxoviruses.

Supplementary Table 1: Bat sampling sites with GPS coordinates

| **Bat Sampling sites** | **GPS coordinates** |
| --- | --- |
| **Centre Region-Yaoundé:** |  |
| Cascades de Mfoundi | E 11^o^30’ 51.13’’ & N 3^o^52’ 5.848’’ |
| Warda – Bois Ste. Anastasie | E 11^o^30’ 52.239’’ & N 3^o^52’ 17.044’’ |
| GP Melen | E 11^o^29’ 33.68112’’ & N 3^o^51’ 46.79136’’ |
| Messa | E 11^o^30’ 42.429’’ & N 3^o^52’ 10.276’’ |
| MINEPAT | E 11^o^30’ 53.863’’ & N 3^o^52’ 7.849’’ |
| **North Region Garoua:** |  |
| Sodecoton-Guider | E 13^o^56’ 37.355’’ & N 9^o^55’ 38.017’’ |
| ENEO-Guider | E 13^o^56’ 42.468’’& N 9^o^55’ 45.3’’ |
| Chefférie Mayo-Oulo | E 13^o^37’ 3.67248’’ & N 9^o^58’ 0.5286’’ |
| Sodecoton-Pitoa | E 13^o^30’ 33.544’’ & N 9^o^23’ 13.002’’ |

*GPS: Global Positioning System, E: East, N: North, GP: Presidential Guard, SODECOTON: La Société de development du Coton, ENEO: Cameroon Electricity Enterprise*

Supplementary Table 2: Rodent sampling sites with GPS coordinates

| **Rodent Sampling sites** | **GPS coordinates** |
| --- | --- |
| **Centre Reggon-Yaounde:** |  |
| Bonas | E 11^o^29’ 39.605’’ & N 3^o^51’ 23.648’’ |
| Emana | E 11^o^31’ 11.678’’ & N 3^o^55’ 43.944’’ |
| Mvog-Betsi | E 11^o^29’ 10.57992’’ & N 3^o^51’ 50.2992’’ |
| Nkolbisson | E 11^o^27’ 0.378’’ & N 3^o^52’ 34.69188’’ |
| Monté Parc | E 11^o^29’ 28.784’’ & N 3^o^52’ 11.662’’ |
| Maison Blanche | E 11^o^28’ 53.07’’ & N 3^o^50’ 37.104’’ |
| Melen | E 11^o^29’ 45.99132’’ & N 3^o^51’ 49.85064’’ |
| Ngoa-Ekelle | E 11^o^30’ 1.92’’ & N 3^o^51’ 17.116’’ |
| Elig-Edzoa | E 11^o^31’ 50.653’’ & N 3^o^53’ 22.222’’ |
| Simbock | E 11^o^28’ 41.22372’’ & N 3^o^48’ 55.73808’’ |
| Manguier | E 11^o^32’ 14.816’’ & N 3^o^54’ 31.385’’ |
| Damase | E 11^o^29’ 16.55808’’ & N 3^o^49’ 28.15212’’ |
| Tsinga | E 11^o^30’ 47.128’’ & N 3^o^53’ 0.074’’ |
| **North Region Garoua:** |  |
| Sodecoton-Guider | E 13^o^ 56’ 37.355’’ & N 9^o^ 55’ 38.017’’ |
| Djamboutou | E 13^o^ 31’ 6.996’’ & N 8^o^ 34’ 51.357’’ |
| Ngalbidje | E 13^o^ 23’ 1.817’’ & N 9^o^ 21’ 13.115’’ |
| Niakira | E 13^o^ 26’ 56.248’’ & N 9^o^ 23’ 2.299’’ |
| Pont Benoue | E 13^o^ 24’ 19.475’’ & N 9^o^ 17’ 48.192’’ |
| Camp Chinois | E 13^o^ 22’ 12.039’’ & N 9^o^ 17’ 49.708’’ |
| Tourouwa | E 12^o^ 58’ 33.085’’ & N 9^o^ 4’ 48.749’’ |

*GPS: Global Positioning System, E: East, N: North, SODECOTON: La Société de development du Coton*

**Supplementary Table 3:** Animals screened by serological analysis and seropositive bats per collection months per sampling region and both regions combined.

|  | **Centre** | | **North** | | **Centre and North** | |
| --- | --- | --- | --- | --- | --- | --- |
| **Month-year** | **bats screened  in serology** | **seropositive bats** | **bats screened  in serology** | **seropositive bats** | **bats screened in serology** | **seropositive  bats** |
| Mar 18 | 34 | 5 | 8 | 0 | 42 | 5 |
| Apr 18 | 0 | 0 | 0 | 0 | 0 | 0 |
| May 18 | 24 | 1 | 0 | 0 | 24 | 1 |
| Jun 18 | 9 | 0 | 27 | 0 | 36 | 5 |
| Jul 18 | 39 | 5 | 30 | 5 | 69 | 10 |
| Aug 18 | 0 | 0 | 0 | 0 | 0 | 0 |
| Sep 18 | 0 | 0 | 0 | 0 | 0 | 0 |
| Oct 18 | 0 | 0 | 0 | 0 | 0 | 0 |
| Nov 18 | 0 | 0 | 0 | 0 | 0 | 0 |
| Dec 18 | 0 | 0 | 0 | 0 | 0 | 0 |
| Jan 19 | 0 | 0 | 0 | 0 | 0 | 0 |
| Feb 19 | 0 | 0 | 0 | 0 | 0 | 0 |
| Mar 19 | 0 | 0 | 86 | 0 | 86 | 0 |
| Apr 19 | 0 | 0 | 0 | 0 | 0 | 0 |
| May 19 | 0 | 0 | 0 | 0 | 0 | 0 |
| Jun 19 | 0 | 0 | 0 | 0 | 0 | 0 |
| Jul 19 | 0 | 0 | 0 | 0 | 0 | 0 |
| Aug 19 | 0 | 0 | 64 | 0 | 64 | 0 |
| Sep 19 | 91 | 2 | 13 | 2 | 104 | 4 |
| Oct 19 | 0 | 0 | 0 | 0 | 0 | 0 |
| Nov 19 | 34 | 1 | 0 | 0 | 34 | 1 |
| Dec 19 | 16 | 0 | 0 | 0 | 16 | 0 |
| Jan 20 | 0 | 0 | 0 | 0 | 0 | 0 |
| Feb 20 | 24 | 0 | 0 | 0 | 24 | 0 |
| Mar 20 | 29 | 3 | 47 | 0 | 76 | 3 |
| Apr 20 | 0 | 0 | 25 | 0 | 25 | 0 |

Supplementary Table 4: Characteristics of rodents and shrews sampled in the Centre and North Regions

| **Centre Region** | **Number of sampled rodents & shrews** | **Proportion of sampled rodents & shrews (%)** | **North Region** | **Number of sampled rodents & shrews** | **Proportion of sampled rodents & shrews (%)** |
| --- | --- | --- | --- | --- | --- |
| **Centre Region -Yaounde** |  |  | **North Region-Garoua** |  |  |
| **Sampling sites** |  |  | **Sampling sites** |  |  |
| Elig Edzoa | 19 | 6.3 | Sodecoton Guider | 7 | 2.3 |
| Damase | 15 | 5 | Djamboutou | 5 | 1.7 |
| Maison Blanche | 17 | 5.7 | Ngalbidje | 7 | 2.3 |
| Ngoa Ekelle | 8 | 2.7 | Niakira | 36 | 12 |
| Monté Parc | 105 | 35 | Pont Benoue | 144 | 48.1 |
| Simbock | 35 | 11.7 | Tourouwa | 100 | 33.3 |
| Mvog-Betsi | 15 | 5 | Camp Chinois | 1 | 0.3 |
| Melen | 1 | 0.3 | / | / | / |
| Nkoabang | 1 | 0.3 | / | / | / |
| Manguier | 7 | 2.3 | / | / | / |
| Tsinga | 57 | 19 | / | / | / |
| Bonas | 12 | 4 | / | / | / |
| Emana | 1 | 0.3 | / | / | / |
| Nkolbisson | 2 | 0.7 | / | / | / |
| GP Melen | 5 | 1.7 | / | / | / |
| **Species** |  |  | **Species** |  |  |
| *Rattus rattus* | 90 | 30 | *Rattus rattus* | 18 | 6 |
| *Rattus norvegicus* | 186 | 62 | *Rattus norvegicus* | 282 | 94 |
| *Mus musculus* | 21 | 7 | / | / | / |
| Shrew | 3 | 1 | / | / | / |
| **Age class** |  |  | **Age class** |  |  |
| Adult | 86 | 28.7 | Adult | 72 | 24 |
| Sub adult | 49 | 16.3 | Sub adult | 53 | 17.7 |
| Juvenile | 165 | 55 | Juvenile | 175 | 58.3 |
| **Gender** |  |  | **Gender** |  |  |
| Male | 64 | 21.3 | Male | 36 | 12 |
| Female | 236 | 78.7 | Female | 264 | 88 |
| **Total** | **300** | **100** | **Total** | **300** | **100** |

*GP: Presidential Guard, SODECOTON: La Société de development du Coton*
